# Genetic Variability Induced by Gamma Rays and Preliminary Results of Low-Cost TILLING on M_2_ Generation of Chickpea (*Cicer arietinum* L.)

**DOI:** 10.3389/fpls.2018.01568

**Published:** 2018-10-31

**Authors:** Wahiba Amri-Tiliouine, Meriem Laouar, Aissa Abdelguerfi, Joanna Jankowicz-Cieslak, Ljupcho Jankuloski, Bradley J. Till

**Affiliations:** ^1^Division of Biotechnology and Plant Breeding, National Institute of Agricultural Research of Algeria, Algiers, Algeria; ^2^Laboratory of Integrative Improvement of Vegetal Productions, Higher National Agronomic School, Algiers, Algeria; ^3^Department of Plant Productions, Higher National Agronomic School, Algiers, Algeria; ^4^Plant Breeding and Genetics Laboratory, Joint FAO/IAEA Division of Nuclear Techniques in Food and Agriculture, IAEA Laboratories Seibersdorf, International Atomic Energy Agency, Vienna, Austria; ^5^Plant Breeding and Genetics Section, Joint FAO/IAEA Division of Nuclear Techniques in Food and Agriculture, Department of Nuclear Sciences and Applications, International Atomic Energy Agency, Vienna, Austria; ^6^Department of Chromosome Biology, University of Vienna, Vienna, Austria

**Keywords:** induced mutations, genetic parameters, path analysis, cluster analysis, yield components, selection, low-cost TILLING, background mutations

## Abstract

In order to increase genetic variability for chickpea improvement, the Kabuli genotype, variety Ghab4, was treated with 280 Grays of gamma rays (Cobalt 60). Field characterization began with the M_2_ generation. A total of 135 M_2_ families were sown in the field resulting in approximately 4,000 plants. Traits related to phenology (days to flowering, days to maturity), plant morphology of vegetative parts (plant height, height of first pod, number of primary branches per plant) and yield (number of seeds per pod, total number of pods per plant, total number of seeds per plant, seed yield and hundred seed weight) were recorded and analyzed to evaluate genetic variability. An evaluation of the efficacy of low-cost TILLING (Targeting Induced Local Lesions IN Genomes) to discover mutations in the M_2_ generation was undertaken. Mutation screening focused on genes involved in resistance to two important diseases of chickpea; *Ascochyta blight* (AB) and *Fusarium wilt* (FW), as well as genes responsible for early flowering. Analysis of variance showed a highly significant difference among mutant families for all studied traits. The higher estimates of genetic parameters (genotypic and phenotypic coefficient of variation, broad sense heritability and genetic advance) were recorded for number of seeds per plant and yield. Total yield was highly significant and positively correlated with number of pods and seeds per plant. Path analysis revealed that the total number of seeds per plant had the highest positive direct effect followed by hundred seed weight parameter. One cluster from nine exhibited the highest mean values for total number of pods and seeds per plant as well as yield per plant. According to Dunnett’s test, 37 M_2_ families superior to the control were determined for five agronomical traits. Pilot experiments with low-cost TILLING show that the seed stock used for mutagenesis is homogeneous and that small mutations do not predominate at the dosage used.

## Introduction

Chickpea (*Cicer arietinum* L.) is the second largest consumed pulse crop of the world, is grown in over 50 countries, and traded across 140 (Gaur et al., 2014). It is the earliest domesticated crop in the Mediterranean and Middle Eastern region (Vavilov, 1951). Chickpea is valued for its nutritive seed composition that is high in protein content and used increasingly as a substitute for animal protein (Shrestha et al., 2011). There are two distinct types of chickpea: Desi (microsperma) and Kabuli (macrosperma). The two types are distinguished by differences in the shape, size and color of seed (Cubero, 1987; Van der Maesen, 1987; Muehlbauer and Rajesh, 2008). The Kabuli type generally has large rounded seeds that are white or cream in color. The Desi type generally has rough seeds with an angular appearance and coloration can vary from light tan to black with all gradations in between (Muehlbauer and Rajesh, 2008). The Kabuli type has been grown traditionally in the Mediterranean basin and Central Asia, while the Desi type has been mainly produced in the Indian subcontinent, East Africa, and Central Asia (Wani et al., 2014).

In Algeria, chickpea is the second important legume crop after faba bean, with the Kabuli type being the most abundant on the market. At the same time, Algeria is the third largest chickpea importer in the world. Therefore, increasing local production will have a positive economic impact. Mutation breeding is an effective and important approach to legume improvement. Induced mutation technique has proved to be successful for improving traits in a wide variety of crops. To date, more than 3,274 varieties in more than 224 plant species derived from mutagenesis programs have been officially released as listed in the FAO/IAEA Mutant Varieties Database (MVD)^1^. Among these, 493 mutant variety pulses are registered, with 21 improved chickpea mutants released for cultivation (Gaur et al., 2007; Saǧel et al., 2009; Mutant Variety Database [MVD], 2016). Mutations can be induced by physical or chemical mutagens. Among physical mutagens, gamma rays are the most frequently used, accounting for 64% of the radiation-induced mutant varieties (Maluszynski et al., 2000; Ahloowalia et al., 2004; Jankowicz-Cieslak and Till, 2015). Induced mutations have a great potential of enhancing genetic variability and thus improving yield in chickpea through effective handling of the mutagenized populations (Wani, 2011). The efficiency of early generation (M_2_) selection in mutation breeding experiments has been reported in various crops (Kozgar, 2014). Therefore, the selection for quantitative traits, such as yield, can and should preferably be carried out in early generations such as an M_2_. This is due to the fact that most of the desired combinations of favorable alleles are likely to be lost in advanced generations due to intensive or even no selection for other traits (Solanki and Sharma, 2001, 2002; Solanki and Rana, 2016). This, however, presupposes that a high frequency of unlinked mutations accumulates at the dosage used for mutagenesis, and that multiple loci contribute to mutant traits. Alternatively, a low frequency of large chromosomal aberrations may accumulate whereby traits may express due to variation at single loci. There is limited data at the genome sequence level to evaluate if expressed traits in released mutant varieties are caused by one or many induced mutations.

Estimates of genetic parameters like phenotypic and genotypic coefficient of variability (PCV, GCV), heritability (H^2^) and genetic advance (GA) for various quantitative traits are useful in designing an effective breeding program (Wani, 2011; Wani et al., 2014; Kozgar, 2014). The genotypic coefficient of variation measures the range of genetic variability shown by the plant trait; however, the GCV alone cannot determine the amount of variation that is heritable (Wani and Khan, 2006; Tabasum et al., 2010; Wani, 2011). Knowledge of heritability is essential for selection-based improvement as it indicates the extent of transmissibility of a character into future generations (Kozgar, 2014). Estimates of heritability alone do not provide an idea about the expected gain in the next generation but have to be considered in conjunction with estimates of genetic advance, the change in mean value between generations (Wani and Khan, 2006; Wani, 2011). Correlation and path coefficients are pre-requisites for improvement of any crop. Knowledge of correlation between yield and its contributing characters are fundamental in establishing guidelines for plant selection. Partitioning of total correlation into direct and indirect effect by path analysis can further improve the effectiveness of selection (Kozgar, 2014).

In recent years, mutagenesis has received great attention as a method for revealing gene function as well as for trait improvement. This is due to developments in the field of molecular biology such as the reverse genetics method called TILLING (Targeting Induced Local Lesions IN Genomes) (McCallum et al., 2000; Till et al., 2006; Kurowska et al., 2011; Hunter et al., 2014). It is an efficient early-screening approach for identification of point mutations in genes of interest. The method is usually applied in combination with chemical mutagenesis because mutation discovery methods are optimized for recovery of single nucleotide and small indel variations, and chemical mutagens produce that spectra of mutations at high density. However, it has been shown that TILLING can be also used in scanning gamma-irradiated mutant populations, as it has been reported that small mutations can be induced under specific gamma irradiation treatments (Sato et al., 2006). Since the first description of TILLING, in the late 1990s, projects have been developed for more than 25 species (Jankowicz-Cieslak et al., 2011; Till et al., 2014; Jankowicz-Cieslak and Till, 2016). The FAO/IAEA Plant Breeding and Genetics Laboratory (PBGL, Seibersdorf, Austria) has developed a series of low-cost and easy to use approaches for the molecular characterization of mutant plant materials suitable for laboratories in developing countries. These methods do not require specialized equipment and do not rely on toxic chemicals (Till et al., 2015; Jankowicz-Cieslak et al., 2017). These include approaches for genomic DNA extraction and extraction of single-strand-specific nucleases and agarose-gel based enzymatic mismatch cleavage assays for mutation discovery (Hofinger et al., 2017; Huynh et al., 2017).

In order to study the effect of irradiation on M_2_ generation, comparisons between M_2_ families and the control variety (non-irradiated) should be carried out. The determination of M_2_ families that are significantly superior to the parent allows selection of improved genotypes. The present study was conducted to evaluate genetic variability induced by gamma rays. Its purpose was to determine the effective agronomic traits with the aim of improving selection in next generations, but also to make selection of genotypes that present an agronomic improvement at a very early generation. Genotypic screening with the use of low-cost TILLING was conducted on the same generation (M_2_), and a panel of genes of interest involved in the major chickpea diseases, *Ascochyta blight* (AB) and *Fusarium wilt* (FW), as well as genes involved in the early flowering pathway.

## Materials and Methods

### Plant Material

Kabuli chickpea variety Ghab 4, a selection from FLIP 93-93C developed by the International Center for Agricultural Research in the Dry Areas (ICARDA) was treated with gamma rays (Cobalt-60), at the Nuclear Research Centre of Algiers (CRNA). The optimum dose of LD50/RD50, corresponding to 280 Grays was applied for the production of high mutant frequency (Amri-Tiliouine et al., unpublished).

### M_1_ and M_2_ Generations

**FIGURE 1:**
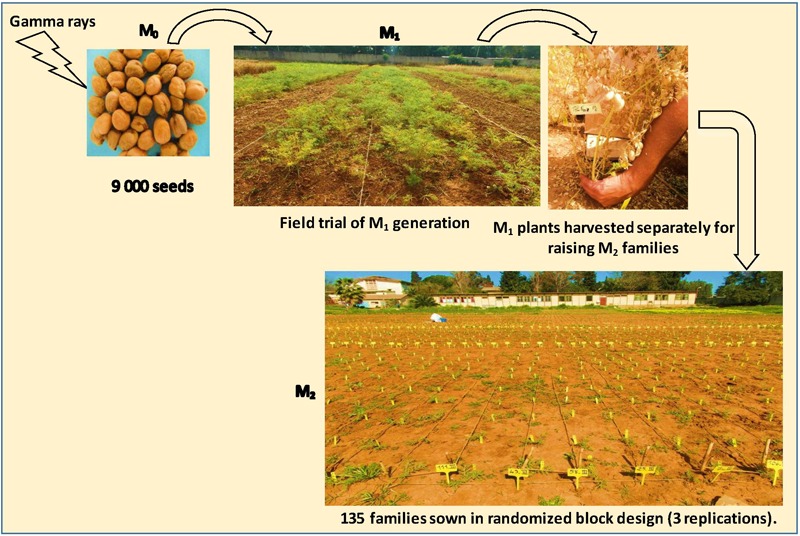
Generation of M_1_ and M_2_ mutant population.

In order to produce the M_1_ generation, the dose of 280 Grays was applied on 9000 healthy and dry (12–13% moisture content) seeds. Untreated seeds were used as control. All seeds were sown in field trial at the experimental station of the Higher National Agronomic School (ENSA), Algiers. The soil characteristic in this field trials is loam clay having 0.07% of nitrogen, 3.6% of organic matter, 10.37 ppm available phosphorus and 7.6 pH. The total precipitation during the trial period from January to July for the two experimental years (2014 and 2015) was 415 mm and 333 mm, respectively. The seed sowing distance was 30 cm × 20 cm with a total area of 700 m^2^. All the M_1_ plants were harvested separately for raising M_2_ generation. Seeds collected from individual M_1_ plants were sown as M_2_ families (Figure 1). A total of 135 individual M_2_ families with 30 seeds per family were sown (50 cm × 35 cm spacing) in randomized block design with three replications. This resulted in 4050 M_2_ plants.

### Data Collection

Observations of various quantitative traits, as indicated in Table 1, were recorded for 4050 M_2_ mutant plants. Data was collected and analyzed to assess the extent of induced genetic variability.

**Table 1:** List of quantitative traits and method of evaluation.

Trait	Denotation	Method of evaluation
Days to flowering	DF (days)	Number of days from sowing to the stage when 50% of plants have begun to flower.
Days to maturity	DM (days)	Number of days from sowing to the stage when over 90% of pods have matured and turned yellow.
Height of first pod	HFP (cm)	Height from the base of the plant to the first pod.
Plant height	HP (cm)	Height from the base of the plant to the tip of last leaf.
Number of primary branches	NPB	Total number of primary branches in a plant.
Number of seeds/pod	NSP	Number of seeds in a pod.
Total number of pods/plant	TPP	Total number of pods per plant.
Total number of seeds/plant	TNS	Total number of seeds per plant.
Seed yield	SY (g/plant)	Weighing the total number of seeds produced in a plant.
Hundred seed weight	HSW (g)	One hundred seeds randomly counted and then weighed.

### Statistical Analysis

The analysis of variance for ten phenotypic characters was carried out using Genstat software version 12.1 (VSN International Ltd., 2009). Genotypic and phenotypic coefficient of variation (GCV, PCV), broad sense heritability (H^2^) and genetic advance as percentage of mean (GA %) were calculated using the following equations:

#### Genotypic Variance (σ^2^g)

$$\sigma^{2}g\;=\;(MSG\;-\;MSE)/r,$$

Where MSG is the mean square of genotypes, MSE is the mean square of error, and *r* is the number of replications.

#### Phenotypic Variance (σ^2^p)

$$\sigma^{2}p\;=\;\sigma 2g\;+\;\sigma^{2}e,$$

Where σ^2^g is genotypic variance and σ^2^e is the mean squares of error.

Error variance (σ^2^e)

$$\sigma^{2}e\;=\;MSE.$$

#### Phenotypic and Genotypic Coefficient of Variation PCV and GCV

The estimates of phenotypic and genotypic coefficient of variation were calculated 190 according to Singh and Choudhary (1985) as follows:

$$\begin{array}{c} PCV\;=\;\frac{\sqrt{\sigma^{2}p}}{\bar{X}}\;\times \;100,\\ GCV\;=\;\frac{\sqrt{\sigma^{2}g}}{\bar{X}}\;\times \;100, \end{array}$$

where σ^2^p is the phenotypic variance, σ^2^g is the genotypic variance and 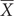 is the mean of the trait. GCV and PCV values were categorized as low (0–10%), moderate (10–20%), and high (20% and above) following Sivasubramanian and MadhavaMenon (1973).

#### Heritability Estimate (Broad Sense) (H^2^%)

$$H^{2}\%\;=\;\frac{\sigma^{2}g}{\sigma^{2}p}\times 100,$$

The heritability percentage was categorized as low (0–30%), moderate (30–60%), and high (≥60%) in accordance with Robinson et al. (1949).

#### Expected and Estimated Genetic Advance (GA)

$$GA\;=\;k\sigma pH^{2},$$

#### The Genetic Advance as Percentage of Mean (GA %)

GA(%) = GA/X × 100,

Was calculated using the method of Assefa et al. (1999) and selection intensity (*k*) was assumed to be 5%; where *k* = 2.06, a constant. σp is the phenotypic standard deviation and H^2^ is the broad sense heritability.

Genetic advance as percentage of mean was categorized as low (0–10%), moderate (10–20%), and high (>20%) (Johnson et al., 1955).

Pearson’s correlation coefficients (*r*) were calculated among all the measured traits using IBM SPSS for Windows Version 20 (2011). Path coefficient analysis permits a critical examination of specific direct and indirect effects of characters and measures the relative importance of each of them in determining the ultimate goal yield. In the present study, we used yield as a dependent variable. This analysis was done using the IBM SPSS AMOS Windows Version 24 (2016) software.

To determine the genetic affinity of M_2_ chickpea families and to perform grouping, clustering analysis was done with R statistical software (eclodist, stats and clv packages) using the Mahalanobis’s distance matrix in Ward2 hierarchical agglomerative clustering method (Murtagh and Legendre, 2014).

In order to identify mutant families significantly superior over the parent (untreated control) the Dunnett’s and Fisher’s (LSD) tests were employed using IBM SPSS AMOS software program Windows Version 24 (2016) and Genstat software version 12.1 (VSN International Ltd., 2009), respectively.

### Development of Low-Cost TILLING

#### Plant Material

Eighty-one samples of chickpea M_2_ putative mutants were chosen for optimization of the low-cost TILLING assays. Non-mutated c.v. Ghab 4 and three different Desi chickpea genotypes were selected as control samples. Seed from the maintained mutant lines will be shared upon request provided sufficient material exists.

#### Genomic DNA Extraction, Quantification, and Normalization

Two different protocols were used for extraction of genomic DNA from chickpea leaves; the low-cost DNA extraction protocol developed by PBGL (Till et al., 2015) and the commercial Qiagen kit. For the low-cost of genomic DNA extraction protocol, leaf material was collected and placed in envelopes and stored in a box containing silica gel (with color indicator). Tissue was ground using a standard vortexer and tungsten carbide beads. The main steps of this protocol involve lysis of the plant material, binding of DNA to silica powder (Celite 545 silica) in the presence of a chaotropic buffer, washing the bound DNA and finally elution of DNA from the silica powder. For the commercial Qiagen kit, leaf material was collected and placed in 2 mL tubes and quick-frozen in liquid nitrogen, either immediately used for DNA isolation or stored at -80°C. Leaves were ground in liquid nitrogen using a Qiagen TissueLyser II (10 sec at 1/30 frequency) and DNA extraction was carried out according to Qiagen Plant Mini Kit protocol.

Extracted DNA was quantified with the use of 1% agarose gel with lambda DNA standards and NanoDrop 1000 Spectrophotometer (Huynh et al., 2017). Genomic DNA samples were then normalized to a common concentration using results obtained from 1% agarose gel electrophoresis and lambda DNA standards and NanoDrop spectroscopy (Huynh et al., 2017). DNA normalization is necessary for pooling samples for TILLING so that each sample in a pool is equally represented in the downstream PCR amplification step.

#### Database Search for Candidate Genes Involved in Resistance to Ascochyta Blight, Fusarium Wilt, and Early Flowering in Chickpea

Primers were designed with the use of NCBI’s database and Primer3 program. The web based programs Coddle http://www.proweb.org/input/ and Primer3 and http://bioinfo.ut.ee/primer3-0.4.0/.

#### Primer Testing and Low-Cost TILLING

Low-cost TILLING with the use of standard agarose gel electrophoresis was adapted for chickpea. The main steps of the process are following: PCR amplification using specific gene primers with pooled template genomic DNA coming from M_2_ plants and parent, followed by enzymatic mismatch cleavage and agarose gel visualization of enzymatic mismatch products (Till et al., 2015).

Different template DNA concentrations were tested (0.1, 0.5, 1, 5, and 10 ng) and a concentration of 1 ng was chosen for subsequent assays. The PCR following PCR mix was used : 82.5 μL H_2_O, 15 μL 10x Ex Taq buffer, 12 μL dNTP mix (2.5 mM), 1.5 μL L primer (10 μM), 1.5 μL R primer (10 μM), 0.38 μL TaKaRa HS taq (5 U/μL). A volume of 7.5 μL DNA and 22.5 μL PCR mix was used.

The thermocycling conditions were: 95°C for 2 min; loop 1 for 8 cycles (94°C for 20 s, 73°C for 30 s, reduce temperature 1°C per cycle, ramp to 72°C at 0.5°C/s, 72°C for 1 min); loop 2 for 45 cycles (94°C for 20 s, 65°C for 30 s, ramp to 72°C at 0.5°C/s, 72°C for 1 min); 72°C for 5 min; 99°C for 10 min; loop 3 for 70 cycles (70°C for 20 s, reduce temperature 0.3°C per cycle; hold at 8°C).

To evaluate performance of different primer combinations, 5 μl of the PCR products was separated on 1.5% agarose gel electrophoresis. Ten microliters of the PCR products were used for enzymatic mismatch cleavage using crude celery juice extract (CJE) containing the nuclease CELI. Different enzyme concentrations were tested, and the following digestion reaction conditions were used for subsequent assays: 10 μl PCR product, 14 μL H_2_O, 4 μL CJE buffer and 2 μL CJE nuclease were mixed and incubated for 15 min at 45°C. Digestions were stopped by cooling the reaction to 8°C and adding 10 μl of 0.25 M EDTA (pH = 8.0). Ten microliters of final reaction were separated by gel electrophoresis on a 1.5% agarose gel stained with ethidium bromide.

#### Sequence Validation of Results Obtained With the Use of Low-Cost TILLING

Sanger sequencing reactions were conducted to evaluate results obtained in enzymatic mismatch cleavage assays. Briefly, pre-sequencing amplification and verification of the PCR product was conducted to ensure sufficient production of a single amplicon. PCR reactions where then purified using one of two methods: enzymatic method (Exonuclease I to remove the excess primer and Shrimp alkaline phosphatase to remove the phosphates from dNTPs) or by using the Qiagen kit (PCR purification kit). Samples were then quantified by fluorimetry (Qubit 2.0) and sent for sequencing to a commercial sequencing facility (LGC genomics^2^).

## Results

### Genetic Variation Among Families

A mutant population of 4050 M_2_ plants representing 135 families was produced from treatment of chickpea seed with 280 Grays gamma irradiation. Visual observations were made on this population to determine the effect of the irradiation treatment. The analysis of data shows that the maximum coefficient of variation (CV%) was observed for total number of seeds/plant and seed yield (58.2% and 58.1%, respectively) followed by total number of pods/plant (55.3%), number of primary branches (31%), hundred seed weight (24.1%), height of first pod (19.3%) and number of seeds/pod (18.4%). Whereas, the coefficient of variation for all other morphological characters was less than 15%. The least coefficient of variation was recorded for days to flowering (2.7%). Analyses of variance showed a very highly significant difference among the families for all the traits studied. The minimum and maximum values for each trait indicated a wide range of differences between genotypes for various characters (Table 2). We observed that the estimated values of corresponding genetic parameters of various quantitative traits in M_2_ generation differ from trait to trait. It is revealed that the greatest genotypic and phenotypic variances were observed for number of seeds per plant, followed by number of pods per plant.

**Table 2:** Estimates of genetic parameters of various quantitative traits in M_2_ generation of Kabuli chickpea.

Traits	Mean	Range	MSG	MSE	σ^2^g	σ^2^p	GCV	PCV	H^2^%	GA	GA %	CV%
Days to flowering (days) (DF)	98.96	95.1–101.4	28.82^∗∗∗^	7.072	7.25	14.32	2.72	3.82	51	3.95	3.99	2.7
Days to maturity (days) (DM)	144.7	139–149.2	115.66^∗∗∗^	20.25	31.80	52.05	3.90	4.99	61	9.08	6.28	3.1
Height of first pod (cm) (HFP)	24.74	17.2–29.1	96.09^∗∗∗^	22.69	24.47	47.16	19.99	27.76	52	7.34	29.67	19.3
Plant height (cm) (PH)	54.97	44.2–59.7	185.58^∗∗∗^	40.58	48.33	88.91	12.65	17.15	54	10.56	19.21	11.6
Number of primary branches (NPB)	3.12	2.2–3.9	2.67^∗∗∗^	0.92	0.58	1.50	24.48	39.30	39	0.98	31.41	31
Number of seeds/pod (NSP)	0.99	0.8–1.3	0.11^∗∗∗^	0.033	0.03	0.06	16.18	24.47	44	0.22	22.05	18.4
Total number of pods/plant (TPP)	53.64	31.9–87.8	3 076.50^∗∗∗^	857.40	739.70	1597.10	50.70	74.50	46	38.13	71.08	55.3
Total number of seeds/plant (TNS)	54.35	30.1–87.2	3 546.00^∗∗∗^	974.70	857.10	1831.80	53.87	78.75	47	41.25	75.90	58.2
Seed yield (g/plant) (SY)	18.33	9.5–28.3	401.80^∗∗∗^	110.40	97.13	207.53	53.77	78.59	47	13.89	75.78	58.1
Hundred seed weight (g) (HSW)	33.54	21.3–39.9	198.99^∗∗∗^	65.45	44.51	109.96	19.89	31.27	40	8.74	26.07	24.1

*^∗∗∗^Significant at 0.001 probability level; MSG, mean square of genotype; MSE, mean square of error; σ^2^g, genotypic variance; σ^2^p, phenotypic variance; GCV, genetic coefficient of variation; PCV, phenotypic coefficient of variation; H^2^, heritability in broad sense; GA, genetic advance at 5 per cent of mean; CV, coefficient of variation.*

### Traits Selection Value

The estimates of PCV in all the traits were higher than the estimates on corresponding GCV. The higher estimates of GCV (>20%) were recorded for number of seeds per plant and seed yield (53.87% and 53.77%, respectively) followed by number of pods per plant (50.70%) and number of primary branches (24.48%). Nevertheless, the smallest GCV was for days to flowering (2.72%). Whereas, the traits classification order obtained is the same for PCV values, the higher PCV values (>20%) were number of seeds per plant and seed yield (78.75% and 78.59%, respectively), followed by number of pods per plant (74.50%) and number of primary branches and hundred seed weight (39.30% and 31.27%, respectively). The smallest PCV was for days to flowering (3.82%).

The same result was obtained for the genetic advance expressed as a percentage of mean. Higher estimates of genetic advance (>30%) were recorded for number of seeds per plant and seed yield (75.90% and 75.78%, respectively) followed by number of pods per plant (71.08%). The lowest value for the genetic advance was for days to flowering (3.99%). The highest value of heritability was recorded for days to maturity (61%). Moderate values between 39 and 54% were observed for all the other traits studied (Table 2). Three quantitative traits, seed yield, number of pods per plant and number of seeds per plant were recorded with higher corresponding PCV (>70%) and higher GCV (>50%), coupled with moderate heritability (46–47%) and higher genetic advance more than 70%. The increased genetic variability for these traits provides great possibility for further selection.

### Relationship Between Traits

Correlation coefficients among morphological traits and yield and its components showed that seed yield was highly significant and positively correlated with number of pods and seeds per plant (Table 3). Positive correlations of seed yield were also observed with plant height, number of seeds per pod, hundred seed weight, and with the number of primary branches. On the other hand, seed yield had a negative correlation with days to flowering and days to maturity and height of first pod. These two traits had a positive correlation with plant height, number of primary branches and number of seeds per pod and negative correlation with days to flowering, days to maturity and height of first pod. Another important negative correlation was observed between plant height and days to flowering (Table 3).

**Table 3:** Correlation coefficients between various quantitative traits in M_2_ generation of chickpea.

	DF	DM	PH	HFP	NPB	NSP	TPP	TNS	SY	HSW
DF	1									
DM	0.312^∗∗^	1								
PH	-0.227^∗∗^	-0.031	1							
HFP	0.085^∗∗^	0.299^∗∗^	0.236^∗∗^	1						
NPB	-0.193^∗∗^	0.035	0.275^∗∗^	0.119^∗∗^	1					
NSP	-0.042^∗^	-0.160^∗∗^	0.149^∗∗^	-0.124^∗∗^	-0.152^∗∗^	1				
TPP	-0.306^∗∗^	-0.324^∗∗^	0.515^∗∗^	-0.266^∗∗^	0.223^∗∗^	0.242^∗∗^	1			
TNS	-0.290^∗∗^	-0.347^∗∗^	0.495^∗∗^	-0.277^∗∗^	0.164^∗∗^	0.399^∗∗^	0.962^∗∗^	1		
SY	-0.294^∗∗^	-0.328^∗∗^	0.519^∗∗^	-0.265^∗∗^	0.150^∗∗^	0.356^∗∗^	0.930^∗∗^	0.946^∗∗^	1	
HSW	-0.033	-0.008	0.137^∗∗^	-0.018	-0.02	0.211^∗∗^	0.065^∗∗^	0.02	0.241^∗∗^	1

*^∗^ and ^∗∗^ indicate significance of values at *P* < 0.05 and 0.01, respectively, according to Pearson correlation.*

**FIGURE 2:**
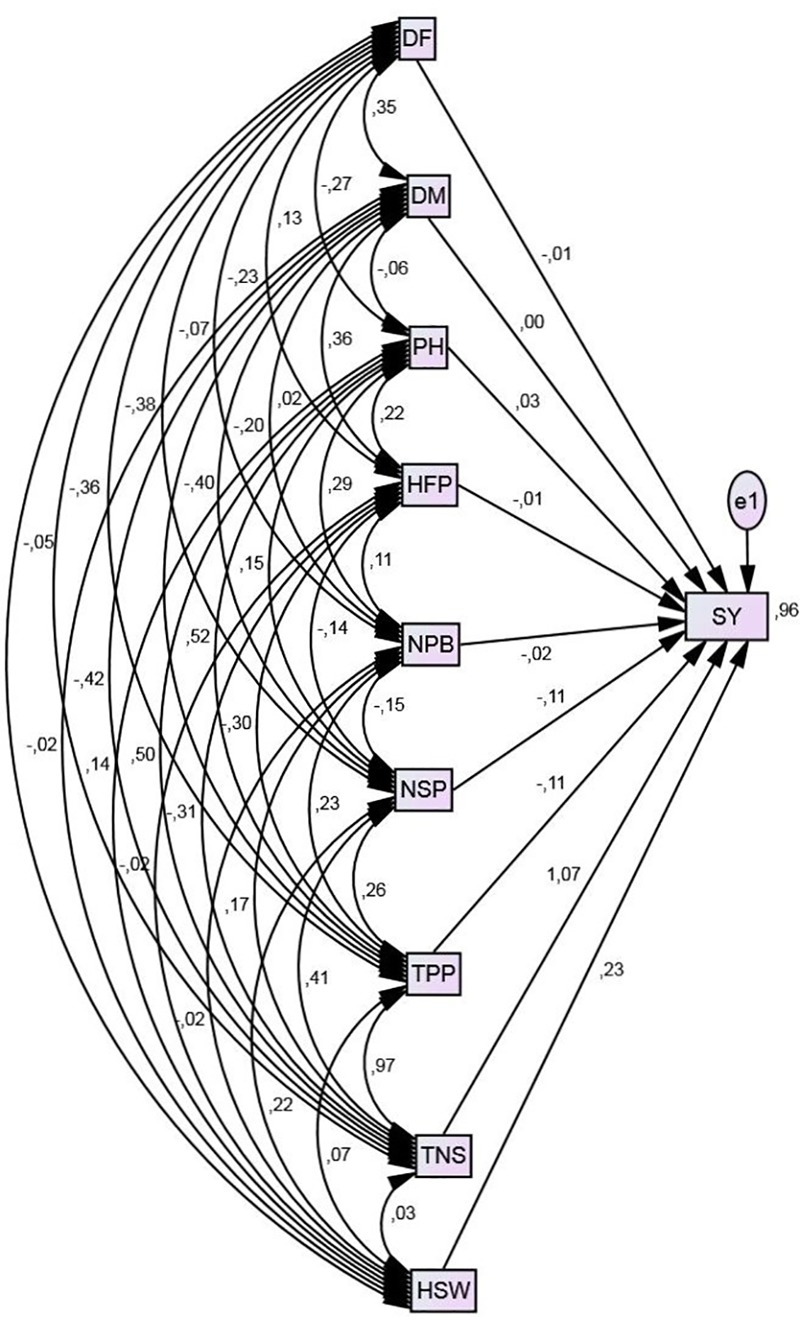
Path diagram representing relationships among different traits and yield in M_2_ generation of chickpea.

Path diagrams showing effect relationships of seeds yield and its components are presented in Figure 2. The direct, indirect and total effects of reproductive traits on seeds yield are presented in Table 4. Path analysis showed that the total number of seeds per plant had the highest positive direct (*p* = 1.07) effect followed by hundred seed weight (*p* = 0.23). Plant height showed negligible positive direct effect on seeds yield per plant (*p* = 0.03). This trait had an important positive indirect effect (*p* = 0.55) through total number of seeds per plant. The number of seeds per pod and total number of pods per plant contributed with negative direct effects (with the same value for both traits *p* = -0.11) on seeds yield. While, date to flowering, height of first pod and number of primary branches showed negligible negative direct effects on yield (*p* = -0.01, *p* = -0.01, and *p* = -0.02, respectively).

**Table 4:** Path analysis showing direct and indirect effect of different traits on seed yield in M_2_ generation of Kabuli chickpea.

Trait	Indirect effect to SY									Total effects		Total correlation to SY
	DF	DM	HP	HFP	NPB	NSP	TPP	TNS	HSW	Direct	Indirect	
DF		0.000	-0.008	-0.001	0.005	0.008	0.042	-0.385	-0.012	-0.01	-0.35	-0.36
DM	-0.0035		-0.002	-0.004	0.000	0.022	0.044	-0,449	-0.005	0.00	-0.40	-0.40
HP	0.0027	0.000		-0.002	-0.006	-0.017	-0.057	0.599	0.032	0.03	0.55	0.59
HFP	-0.0013	0.000	0.007		-0.002	0.015	0.033	0.332	-0.005	-0.01	0.38	0.37
NPB	0.0023	0.000	0.009	-0.001		0.017	-0.025	0.182	-0.005	-0.02	0.18	0.16
NSP	0.0007	0.000	0.005	0.001	0.003		-0.029	0.439	0.051	-0.11	0.47	0.37
TPP	0.0038	0.000	0.016	0.003	-0.005	-0.029		1.038	0.016	-0.11	1.04	0.93
TNS	0.0036	0.000	0.015	-0.003	-0.003	-0.045	-0.107		0.007	1.07	-0.13	0.94
HSW	0.0005	0.000	0.004	0.000	0.000	-0.024	-0.008	0.032		0.23	0.01	0.24

Regarding the indirect effects on seed yield per plant, it was found that total pods per plant showed maximum positive indirect effect (*p* = 1.04) followed by plant height (*p* = 0.55), number of seeds per pod (0.47), height of first pod (0.38), number of primary branches (*p* = 0.18) and hundred seed weight (*p* = 0.01). Whereas, date to flowering (*p* = -0.35), date to maturity (*p* = -0.40) and total number of seeds per plant (*p* = -0.13) showed negative indirect effect on seeds yield. The indirect effects on seeds yield of the majority of studied traits is mainly through total number of seeds per plant.

### M_2_ Population Structure

**FIGURE 3:**
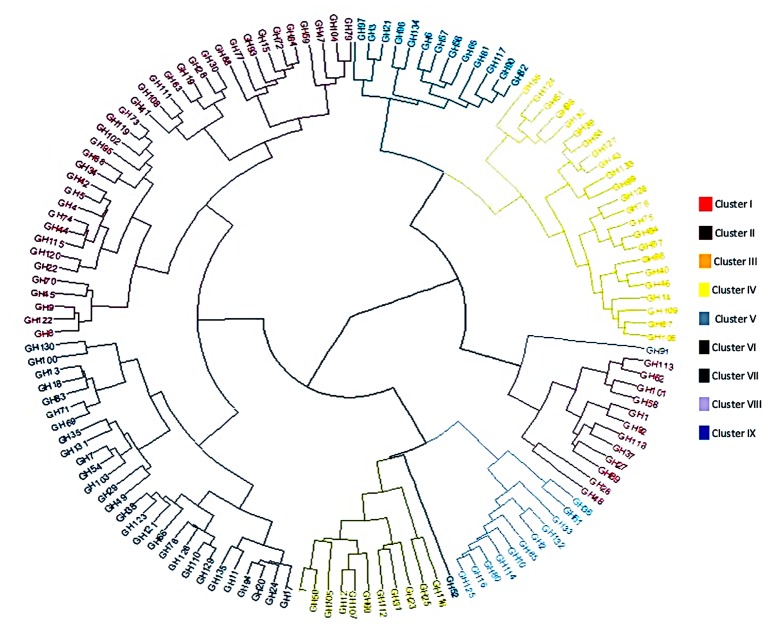
Phenogram showing hierarchical clustering of 135 M_2_ chickpea families grouped in nine clusters. Mahalanobis’s distance used for clustering based on 10 quantitative traits.

Clustering analysis of the phenotypic performance in ten quantitative traits using the Mahalanobis’s distance in Ward’s hierarchical agglomerative clustering method grouped the 135 M_2_ chickpea families in nine clusters (Figure 3). Among the nine clusters, cluster II had the largest number of families (36), followed by clusters V and IV which had 28 and 23 families, respectively. Clusters IX and I had, respectively, 13 and 12 families. Clusters III and VI grouped each one 11 families; whereas, clusters VII and VIII contained only one family each. The non-mutated control belongs to the cluster VI.

The cluster mean for each character is presented in Table 5. Results showed that highest mean value for days to flowering (100.02) and days to maturity (147.12) was observed in cluster IX, while the lowest mean values for these characters was observed in cluster VIII (95.14 and 140.28, respectively). Cluster II exhibited the highest mean values for number of primary branches (3.29) and plant height (56.25 cm), however, the lowest mean values for these characters were observed in cluster VIII (2.46 and 44.21 cm, respectively). Cluster VI exhibited the highest mean values for total number of pods (71.30) and seeds per plant (73.69) and yield per plant (23.22 g). The low mean values for total number of pods (40.36) and seeds per plant (40.77) were found in cluster IX. While, for yield per plant were found in cluster VIII (13.74 g) followed by cluster IX (13.89 g). Overall results indicated that maximum mean values for most of the yield contributing characters were found in cluster VI.

**Table 5:** Cluster means of 135 M_2_ chickpea families for the various quantitative traits.

Traits	Cluster I	Cluster II	Cluster III	Cluster IV	Cluster V	Cluster^∗^ VI	Cluster VII	Cluster VIII	Cluster IX
Days to flowering	98.59	98.31	98.68	98.94	99.13	97.52	99.93	95.14	100.02
Days to maturity	143.63	144.16	142.92	145.45	145.93	142.17	146.15	140.28	147.12
Plant height (cm)	51.33	56.25	55.88	53.94	56.02	55.09	55.28	44.21	53.52
Height of first pod (cm)	22.86	24.91	24.39	25.14	25.58	22.54	27.48	19.58	26.36
Number of primary branches	2.92	3.29	2.63	3.26	3.03	3.21	3.28	2.46	3.06
Number of seeds/pod	0.97	0.98	1.05	0.95	0.99	1.04	1.32	0.78	1.00
Total number of pods/plant	51.75	59.02	59.50	43.10	51.75	71.30	50.96	49.63	40.36
Total number of seeds/plant	52.16	59.10	62.76	42.97	51.63	73.96	72.72	46.83	40.77
Seed yield (g)	16.87	20.23	22.22	14.16	18.17	23.22	17.36	13.74	13.89
Hundred seed weight (g)	32.71	33.98	35.19	31.79	35.19	31.87	21.33	26.71	34.16

*^∗^Control was represented in this cluster.*

### Selection of Promising M_2_ Mutant

Results of Dunnett’s test for the ten traits studied are listed in Table 6. These data indicated variability among the chickpea families. For all the traits there were families with mean values significantly different from the control Ghab 4. According to Dunnett’s test, 37 M_2_ families superior to the control were determined for five agronomical traits [days to flowering, days to maturity, height of first pod (cm), number of primary branches and number of seeds/pod].

**Table 6:** Means comparison with that of the control (Dunnett’s test) of 135 M_2_ families.

Characters	Number of families inferior	Number of families superior
Days to flowering	1	18
Days to maturity	1	13
Plant height (cm)	22	0
Height of first pod (cm)	2	4
Number of primary branches	0	1
Number of seeds/pod	8	1
Total number of pods/plant	23	0
Total number of seeds/plant	36	0
Seed yield (g)	55	0
Hundred seed weight (g)	4	0

Considering the number of days to flowering and the number of days to maturity, 18 and 13 families, respectively, presented mean values higher than that of the control. Only one family showed mean values lower than control, while the others remained in the same class as the control. For the height of first pod, four mutant families showed means scored above the control. For number of primary branches and number of seeds per pod, one mutant family showed a superior performance in comparison to the control. While for the following characters: total number of pods per plant, total number of seeds per plant, seed yield and hundred seed weight, no significant positive differences were identified with Dennett’s test.

Whereas the groupings of averages according to Fisher’s test (LSD) analysis for these traits showed significant differences. Considering the total number of pods per plant, nine families, presented mean values higher than the control. For the total number of seeds per plant and the yield, four families (GH105, GH50, GH112, and GH60) and two families (GH105 and GH50), respectively, showed means above the control. Finally, 30 families presented hundred seed weight higher than that of the control. For the plant height according to Fisher’s test (LSD) seven families (GH108, GH50, GH66, GH38, GH111, GH28, and GH41) presented mean values higher than that of the control.

### Development of Low-Cost TILLING for Mutation Discovery in Chickpea

**FIGURE 4:**
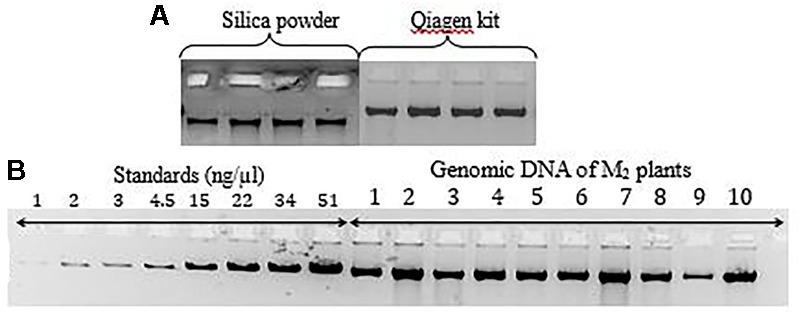
**(A)** Qualitative comparison of DNA isolated using two different protocols (low cost DNA extraction protocol with silica powder and the commercial Qiagen kit) on agarose gel electrophoresis. **(B)** Quantification and qualitative verification of DNA obtained using standards and visualization 1% agarose gel electrophoresis.

An important step in the development of low-cost TILLING is the production of genomic DNA that is of high molecular weight and of sufficient quantity for planned assays. We first sought to test molecular weight of genomic DNA produced using a home-made low-cost extraction protocol versus a commercial Qiagen kit. Agarose gel analysis shows that both methods produce a single high molecular weight band (Figure 4A). The concentration of genomic DNA was then estimated using agarose gel analysis by comparison with standards of known concentration (Figure 4B). From this we conclude that both extraction methods produce genomic DNA of sufficient quality and quantity for low-cost TILLING.

### Database Search for Candidate Genes Involved in Resistance to Ascochyta Blight, Fusarium Wilt, and Early Flowering in Chickpea

Sequences of candidate genes were obtained from the NCBI database. Primer design was carried out using the CODDLE tool to choose optimal gene regions for TILLING. This tool incorporates the Primer3 software for primer design. Twelve primer pairs were designed (Table 7) for the target genes.

**Table 7:** Chickpea primers name, sequences, and PCR product size.

Reference	Primer name	Primer sequence	Product size (bp)
A1	ca_erlp_1F	TCAATTCAAGTCCTCGATCTGCATCC	1399
A1	ca_erlp_1R	GCACATTGCCGACTTTCAACATCGT	
A2	ca_erlp_2F	CGGTCCTTCCCCTCTTTCCCTCTCT	1424
A2	ca_erlp_2R	GAGACTGCTGCAACGCTCGGTTCT	
A3	ca_erlp_3F	GGAGGGCCTAAGCTTCAACATGTGC	1385
A3	ca_erlp_3R	GGCCAGTTAGGGCCATTGAACTTCC	
A4	ca_erlp2_1F	ATGCTTTTTGGGGTTAGGTGGGTGT	1486
A4	ca_erlp2_1R	GGAACGGTGCTTCATAGCGGTTACCT	
A5	ca_erlp2_2F	CGGTCCTTCCCCTCTTTCCCTCTCT	1446
A5	ca_erlp2_2R	CTGGCCATCATTGCGTTCTTCTGAG	
A6	ca_erlp2_3F	GGGTCCATTTTTCGAATCAGGTTGG	1453
A6	ca_erlp2_3R	A GCAGCTTCCCTCAACATGGAATGG	
A7	ca_efg_1F	GAGTATCCGCATCCACCAAGGCAAC	1306
A7	ca_efg_1R	TGTCGCTCCCAAGTCCTAACATCCTG	
A8	ca_efg2_2F	TGCACGATGACCCCTGGATGTATGGT	1177
A8	ca_efg2_2R	GGCGACTGATTGAAACACCAGGGACC	
A9	ca_must2_1F	GGAATCAATCCAACCCAAACCGAAA	1478
A9	ca_must2_1R	CGACTGCAGCATTGGTTTCTTCGAG	
A10	ca_must2_2F	TGCATCTATGATTCAGGCTGCATTTGA	1500
A10	ca_must2_2R	GTCCGGGAAGAACGAAACGCATGTA	
A11	ca_must2_3F	AAATGACGAGTTGCAGCGAGCAAGA	1482
A11	ca_must2_3R	GTGTTGCAGCAAGGTCTTCCACCAC	
A12	ca_must2_4F	GCTGCTCCTTCAAGAGGGTTTGTTCC	1002
A12	ca_must2_4R	CTTGGGTTTGAGGGGGTGTTGGAAT	

### Polymerase Chain Reaction (PCR)

**FIGURE 5:**
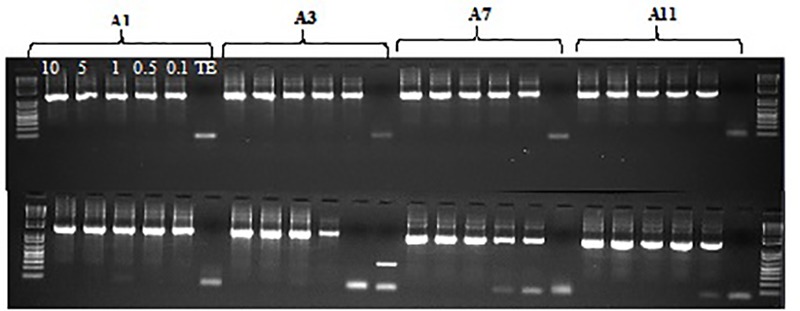
Agarose gel evaluation of PCR primers test (four primers A1, A3, A7, A11) with five different concentrations of genomic DNA 10, 5, 1, 0.5, 0.1 ng/μl and TE control, DNA of parent Ghab 4 (comb 1), and DNA of M_2_ plants (comb 2).

Chosen primer pairs were then tested for their ability to produce a single amplification product of the expected molecular weight. The verification of the twelve primer pairs for gene regions involved in resistance to Ascochyta blight, Fusarium wilt, and early flowering in Chickpea was performed with DNA obtained from parent Ghab 4 and M_2_ putative mutant. Four different genomic DNA concentrations were evaluated. After amplification, PCR reactions were visualized on the 1% agarose gel (Figure 5 and not shown). From these tests, 10 primer pairs were chosen for low-cost TILLING assays (Table 8).

**Table 8:** Result of primer screening with genomic DNA of chickpea Ghab 4 variety and some M_2_ samples.

Reference	Primer name	Amplification
A1	ca_erlp_1	**+**
A2	ca_erlp_2	**+**
A3	ca_erlp_3	**+**
A4	ca_erlp2_1	-
A5	ca_erlp2_2	**+**
A6	ca_erlp2_3	-
A7	ca_efg_1	**+**
A8	ca_efg2_2	**+**
A9	ca_must2_1	**+**
A10	ca_must2_2	**+**
A11	ca_must2_3	**+**
A12	ca_must2_4	**+**

Low-cost TILLING assays utilize a crude enzyme mixture that recognizes and cleaves heteroduplex DNA formed by denaturation and annealing of PCR products of different sequence. As such, the assay will not detect homozygous mutations if screening individual M_2_ plants. To overcome this, an equal amount of parental (non-mutated) genomic DNA is mixed with each sample prior to PCR amplification. This way both homozygous and heterozygous mutations can be detected. TILLING assays were performed with the 10 selected primers and 81 M_2_ genomic DNAs mixed with wild type. Products of enzymatic digestion were visualized on 1.5% agarose gel.

**FIGURE 6:**
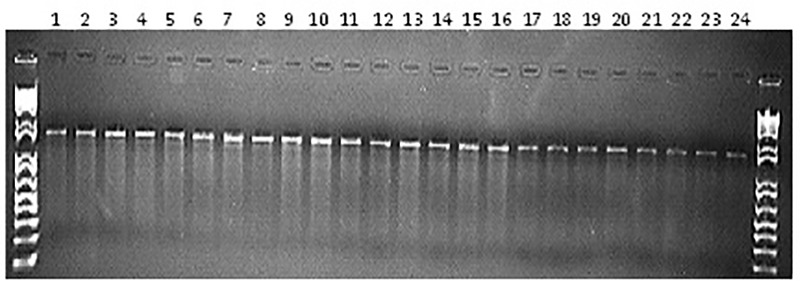
Agarose gel evaluation of enzymatic digestion using A2 (ca_erlp_2) primer with DNA of M_2_ plants (from 1 to 24).

**FIGURE 7:**
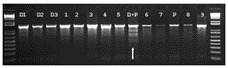
Agarose gel evaluation of enzymatic digestion using A3 (ca_erlp_3) primers with genomic DNA mix, with the DNA of the following samples: 1, 2, 3, 4, 5, 6, 7, 8, 9: M2 plants with Ghab4 parent, D1: Desi genotype n°1, D2: Desi genotype n°2, D3: Desi genotype n°3, P: Ghab 4, D+P: Three Desi genotypes with Ghab4 parent.

No nucleotide variation was observed in these assays (Figure 6 and not shown). To ensure that lack of recovery of mutations was not due to assay failure, we performed an experiment with mixtures of the Desi genotype from Bangladesh and the Ghab4 parent from Algeria, with the expectation that natural SNPs would be present. This serves as a positive control for the assay. Indeed, this experiment showed a high level of polymorphism in the mixture (Figure 7). Observed nucleotide variation was validated by Sanger sequencing (not shown). From this we conclude that low-cost TILLING is suitable for chickpea, and the seed stock used for mutagenesis of Ghab4 is highly homozygous as well as that the frequency of induced small mutations is likely less than one mutation per million base pairs (13,599 unique bp screened × 81 samples).

## Discussion

Genetic variability is a pre-requisite to the selection of superior genotypes in any crop. Thousands of years of human selection, however, has led to the loss of potentially important allelic variation. Strategies that aim to increase genetic variation within a crop species can dramatically improve the efficiency of the breeding process. Unlike wide-crosses, mutagenesis can be applied directly to the elite cultivar without unwanted linkage drag. In this study, we used gamma irradiation on Algerian chickpeas. The success of this approach relies on the application of an irradiation dosage that induces sufficient variation while still maintaining fertility. Variation can be measured phenotypically and genotypically. While genotypic analysis allows precise estimations of DNA variation, phenotypic analysis provides important data on the expression of useful traits and their heritability.

### Variability and Performance of Superior Mutants for Breeding

The mutant population showed very highly significant differences among families for all traits scored. Nine families exhibited the highest mean values for total number of pods and seeds per plant as well as yield per plant. The maximum of variability was observed for the component of yield (pods per plant, number of seeds per plant and yield) for which the coefficient of variation was very high. This variation was not only genetic but also was influenced by the growing environment. Malik et al. (1988); Arshad et al. (2004), Barshile and Apparao (2009), and Saki et al. (2009) have reported similar results in chickpea. Similar results in the M_2_ generation of mung bean (*Vigna radiata* L.) and black gram (*Vigna mungo* L. Hepper) have been reported (Roychowdhury et al., 2012; Usharani and Ananda Kumar, 2016). High genetic advance expressed as a percentage of mean for seed yield per plant and number of pods per plant observed in the present study was also reported by Raval (2001); Parshuram et al. (2003), and Vaghela et al. (2009).

High heritability with moderate genetic gain observed in the mutant population indicates that the characters were governed by additive gene interactions. This is interesting to consider as traits important to breeding may be due to mutation of more than one locus. This has implications when considering reverse-genetic approaches such as TILLING or CRISPR. Further, higher estimates of genetic components (GCV, PCV, H^2^, and GA) noted in this study for seed yield, pods per plant and seeds per plant have also been reported by Adhikari and Pandey (1982); Arora (1991), Wani and Khan (2006); Yimram et al. (2009), Degefa et al. (2014), and Raturi et al. (2015) in different crop legumes (chickpea and mung bean).

### Best Traits for Efficient Selection

Knowledge of the correlation between the characters of the plant is helpful in the selection of performance improvement traits. The positive and significant relationship of the number of pods and seeds per plant with seed yield indicates the importance of these traits in determining seed yield. Similar positive correlations were reported by Hassan et al. (2005). The result concerning the positive correlations of seed yield observed with plant height, number of seeds per pod, hundred seed weight, and with the number of primary branches supplements the finding of Malik et al. (1987); Khan et al. (1989), Arshad et al. (2004), and Islam et al. (2008). The study of Mallu et al. (2015) in chickpea showed the same negative correlation obtained in this study of seed yield with days to 50% flowering and days to 50% podding. This indicated the earliest families are the most productive. Our findings from path analysis for the total number of seeds per plant, hundred seed weight and plant are in accordance with previous reports of Hassan et al. (2005) in mutant chickpea lines, except that for the plant height, which had maximum positive and indirect effect via hundred seed weight not via total number of seeds per plant. Khan and Qureshi (2001) stated that hundred seed weight had direct positive effect on seed yield per plant. However, the highest positive direct effect of the number of seeds per plant on seed yield confirms the findings of Samad et al. (2014) in chickpea.

Correlation analysis showed consequently a positive and significant relationship between the number of pods per plant and seed yield, but path analysis showed that its direct effect was negative. So, the positive relationship was because of its positive indirect effects via the number of seeds per plant. Results observed in this study have also been reported by Hassan et al. (2005). Whereas, correlation coupled with path coefficient analysis revealed that the number of seeds per plant had a direct relationship with seed yield. Our study suggests that for improvement of seed yield the selection upon number of pods per plant and number of seeds per plant will provide positive results. Clustering analysis based on ten quantitative traits shows a high level of phenological diversity in the M_2_ chickpea families.

Among the various traits studied in chickpea, the most important in breeding programs are seed yield, height of plants and of first pods and seed mass. However, the use of tall erect types would facilitate harvesting, both by hand and by mechanical methods (Patil, 2013). According to Chaturvedi et al. (2016) the development of chickpea varieties for their amenability to machine harvesting is one of the potential strategies to reduce cost of cultivation. The result obtained for the plant height was consistent with that obtained by Luz et al. (2016) in rice mutant families. Luz et al. (2016) has reported that among the characters scored, plant height was the most affected by the mutagen. The reduction of plant height was also reported by Khan et al. (2005), Wani (2011), and (Kozgar, 2014) in chickpea mutant families.

### Low-Cost TILLING for Reverse-Genetics

Molecular biology assays can be incorporated into distinct steps of the mutation breeding process to improve its efficiency (Jankowicz-Cieslak and Till, 2015). Screening the M_2_ generation is used to evaluate the efficiency of mutation induction and also as a reverse-genetic tool to target mutations in specific genes. An ideal assay would be able to recover all types of induced variation including SNPs, large and small indels, copy number variation and chromosomal rearrangements. This can be accomplished but requires a combination of short and long-read whole genome sequencing at a depth of coverage that is cost prohibitive for many laboratories.

An alternative approach is to use low-cost assays that enable a quick evaluation of the mutant population. The first step to any molecular assay employing genomic DNA is the extraction of high quality DNA. We show in this work that low-cost, non-toxic genomic DNA extraction is suitable for chickpea. This is advantageous to alternative methods because leaf material is collected at room temperature and dried using reusable silica gel. Thus, the expense and hazard of liquid nitrogen and the need for continuously powered -80°C freezers is avoided. Further, the assay does not use organic phase separation and so toxic chemicals are avoided. Finally, assay cost can be reduced nearly 10-fold compared to some commercial kits, and DNA can be produced for less than 20 United States cents per sample (Huynh et al., 2013).

We further show that self-extracted nuclease and agarose gel electrophoresis is suitable for discovery of nucleotide variation in chickpea. Nucleotide polymorphisms were easily identified in mixtures of diverse chickpea genotypes. This provides a useful positive control when screening for induced mutations, as the frequency of induced mutation events is expected to be low and recovery of induced mutations will be rare. Indeed, when screening 81 samples with 10 primer pairs we found no evidence of induced small nucleotide variation. From this we conclude that the seed used for mutagenesis was highly homogeneous and homozygous. The starting material for mutagenesis for mutation breeding and reverse-genetics is ideally isogenic. Thus, the low-cost TILLING assay we developed provides a useful check of the seed stock used for mutagenesis. This work also provides important information on the induction of mutations. In this assay, we screened approximately one million base pairs, and so we estimate the small mutation frequency to be below 1 per one million bp.

The largest data sets on mutation frequency come from chemical mutagenesis based TILLING experiments. Chemical mutagens such as EMS (ethyl methyl sulfonate) are known to induce primarily single point mutations. The expectation is that SNP mutations can accumulate to higher levels that other mutation types because a high percentage of SNP mutations will have no negative effect on gene function. Reported mutation densities in diploids has ranged between 1 mutation per 150 kb to over one mutation per one million base pairs (Till et al., 2018). In chickpea, mutation frequency was estimated by TILLING platform through the analysis of 768 M_2_ progenies (obtained with 0.2% EMS mutagen) using 20 targets comprising genomic DNA and cDNA sequences. The frequency of mutations determined was one per 165 kb. This frequency is ∼1.6× higher than reported in Arabidopsis (Muehlbauer and Rajesh, 2008).

The spectrum of mutations induced by gamma irradiation is, however, more diverse. Recent advances in next generation sequencing are allowing a more comprehensive measurement of gamma-induced mutation events. For example, large insertion and deletion events predominate in gamma irradiated maize and poplar, while seed irradiation of rice resulted in an abundance of SNP and small indel variation (Yuan et al., 2014; Henry et al., 2015; Li et al., 2016). The low-cost TILLING assay that we describe has an ascertainment bias in that only point mutations and small insertions-and deletions (up to approximately 50 nt) (Till, 2014) will be efficiently recovered. Using similar assays, Sato et al. (2006) estimated the rate of mutation induced by gamma rays in rice to be one mutation per 6,190 kb. While, Hwang et al. (2016) report that the average number of mutations per gene was 1/492 kb in M_2_ rice irradiated lines and the percentage of mutation sites per total sequence was 0.67.

It is likely that fewer mutations will accumulate in combinations of genotypes and dosages where the induction of large indels predominates. This is because essential genes will be quickly knocked out leading to lethality. Based on current data, this makes the evaluation of gamma irradiated populations more challenging compared to chemically mutagenized populations. Certain combinations of dosage and genotype may produce a higher or lower frequency of small mutations. In this context, our observed density of <1 small mutation per million base pairs fits within observations for gamma irradiated crops. It remains possible that our population harbors more large variation that cannot be observed by low-cost TILLING. A low-coverage whole genome sequencing approach (LCWGS) can be applied to discover and catalog such variation in plants (Henry et al., 2015; Datta et al., 2018). As sequencing prices drop, this approach becomes feasible for more laboratories. LCWGS, however, is not suitable for accurate SNP calling, and so a dual screening approach with low-cost TILLING can be considered for characterizing future populations.

This work highlights the efficacy of employing both phenotypic and genotypic screening approaches. We conclude that new variation has been induced in chickpea by treatment of seed irradiation through phenotypic observations. This suggests that the population is suitable for breeding. We have also adapted low-cost mutation discovery assays for chickpea and show these are suitable for recovery of small nucleotide variations. We further conclude that larger induced mutations may be present in this population at sufficient frequency to warrant evaluation by whole genome sequencing. Characterization of the mutant population is ongoing. The promising mutants are currently being tested under controlled conditions (rainfed and irrigated) with the use of ^15^N-labeled fertilizer to evaluate their drought tolerance. The goal of this evaluation is to identify chickpea genotypes expressing high agronomic nitrogen and water use efficiency. The physiological traits analysis and measurements utilizing ^15^N and ^13^C, are in progress. Low-cost TILLING assays on the whole M_2_ population are also ongoing and should be finalized by the beginning of 2019. Further evaluations should shed light on the link between induced genotypic variation and the observed expressed phenotypes. This will provide useful information on gene function and also provide markers to facilitate breeding. Collaborations with interested researchers on this mutant population are highly encouraged, particularly on topics surrounding food technology.

## Author Contributions

ML, WA-T, and AA conceived the study. WA-T conducted the experiments. WA-T and ML analyzed the data. WA-T and ML wrote the manuscript. JJ-C and BT contributed to data analysis and manuscript preparation of the genotyping sections of this manuscript. AA, LJ and contributed to writing. All authors read and approved the manuscript.

## Conflict of Interest Statement

The authors declare that the research was conducted in the absence of any commercial or financial relationships that could be construed as a potential conflict of interest.

## Footnotes

1 http://mvd.iaea.org

2 https://shop.lgcgenomics.com/
